# Both the apoptotic suicide pathway and phagocytosis are required for a programmed cell death in *Caenorhabditis elegans*

**DOI:** 10.1186/s12915-016-0262-5

**Published:** 2016-05-16

**Authors:** Holly L. Johnsen, H. Robert Horvitz

**Affiliations:** Howard Hughes Medical Institute, Department of Biology, Massachusetts Institute of Technology, 77 Massachusetts Avenue, Cambridge, MA 02139 USA

**Keywords:** Programmed cell death, Apoptosis, Phagocytosis, Engulfment, Phagoptosis, *C. elegans*

## Abstract

**Background:**

Programmed cell deaths in the nematode *Caenorhabditis elegans* are generally considered suicides. Dying cells are engulfed by neighboring cells in a process of phagocytosis. To better understand the interaction between the engulfment and death processes, we analyzed B.al/rapaav cell death, which has been previously described as engulfment-dependent and hence as a possible murder.

**Results:**

We found that B.al/rapaav is resistant to caspase-pathway activation: the caspase-mediated suicide pathway initiates the cell-death process but is insufficient to cause B.al/rapaav death without the subsequent assistance of engulfment. When the engulfing cell P12.pa is absent, other typically non-phagocytic cells can display cryptic engulfment potential and facilitate this death.

**Conclusions:**

We term this death an “assisted suicide” and propose that assisted suicides likely occur in other organisms. The study of assisted suicides might provide insight into non-cell autonomous influences on cell death. Understanding the mechanism that causes B.al/rapaav to be resistant to activation of the caspase pathway might reveal the basis of differences in the sensitivity to apoptotic stimuli of tumor and normal cells, a key issue in the field of cancer therapeutics.

**Electronic supplementary material:**

The online version of this article (doi:10.1186/s12915-016-0262-5) contains supplementary material, which is available to authorized users.

## Background

Programmed cell death, often referred to as apoptosis, is an evolutionarily conserved process that plays critical roles in normal animal development and tissue homeostasis; dysregulation of programmed cell death can cause disorders as diverse as cancer, autoimmune disease and retinal degeneration [1, 2]. The genetic pathway that controls programmed cell death in *C. elegans* is known and evolutionarily conserved. Both pro-death and pro-survival proteins are likely present in most if not all cells [3]. The decision of a cell to die is generally made at the level of the transcriptional control of the pro-apoptotic gene *egl-1* [4]. When the EGL-1 BH3 family protein is produced, it disrupts the cell-protective interaction between the anti-apoptotic CED-9 Bcl-2-family protein and the CED-4 Apaf-1-like protein, releasing CED-4 to activate the pro-apoptotic caspase CED-3 [3–8]. These genes function cell-autonomously in cell death [3, 9], and *C. elegans* cell deaths have generally been considered to be suicides.

**Fig. 1:**
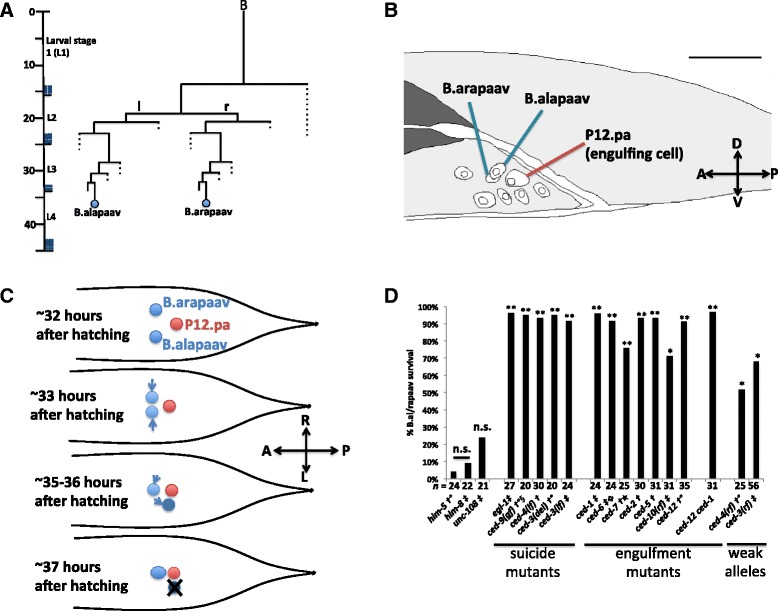
B.alapaav and B.arapaav are left-right homologs and B.al/rapaav death is dependent on engulfment and suicide genes. **a** The B.alapaav and B.arapaav cell lineages. Figure adapted from Sulston et al. [16]. **b** B.alapaav and B.arapaav are located close to each other and to the engulfing cell P12.pa in the developing male tail. Cell nuclei were traced from a DIC image of an otherwise wild-type male of genotype *nIs343[P*_*egl-1*_ *::4xNLS::gfp]; him-8* just before the fourth larval stage, about 34 hours after hatching. Scale bar: 10 μm. DIC Z-stack of this animal is available as Additional file 1: Movie 1. **c** A schematic of the movements of B.alapaav and B.arapaav (blue) as viewed from above the animal. At the time of their generation, B.alapaav and B.arapaav are located to the left and right sides of the rectum. They move closer to the midline, ventral to the rectum. Eventually, the B.al/rapaav homolog will move closer to the midline, and B.al/rapaav typically moves slightly posterior and further from the midline. The B.al/rapaav homolog survives, and B.al/rapaav will undergo programmed cell death. In this diagram, B.alapaav is the dying B.al/rapaav and B.arapaav is the surviving B.al/rapaav homolog. **d** The percentages of late fourth larval stage males with a living *P*_*egl-1*_ *::4xNLS::gfp*-expressing secondary B.al/rapaav (i.e. B.al/rapaav was neither absent nor fully-refractile by DIC, see main text). All genotypes include *nIs343* and some also contain †: *him-5(e1490)*, ‡: *him-8*, °: *nIs349*, §: *lon-1(e1820) dpy-17*, : *lon-1(e185)*, ★: *unc-32*. n.s.: *P* > 0.5, *: *P* < 0.005, **: *P* < 10^−5^

Each dying cell is engulfed by a neighboring cell. In mutants deficient in the engulfment process, the vast majority of cells still undergo programmed cell death, but the cell corpses persist unengulfed and only slowly degrade [10–12]. Only a very small number of cells stochastically fail to die in engulfment-defective mutants [12]. Thus, engulfment is not generally required for the death process. Engulfment can promote the deaths of sensitized cells (e.g., cells that would normally die but instead sometimes survive in mutants with reduced *ced-3* function), possibly by preventing injured cells from recovering [12–14]. By contrast, there is one cell death in *C. elegans* that has been speculated to be a murder, based on the observation that if the engulfing cell is killed using laser microsurgery, cell death can be prevented. Specifically, the two left-right homologous cells B.alapaav and B.arapaav in the tail of the third-larval stage male constitute a developmental equivalence group [15, 16]: either can survive (the primary fate), while the other undergoes programmed cell death and is engulfed by a neighboring cell, P12.pa (the secondary fate) (Fig. 1a–c and Additional file 1: Movie 1). This death occurs during the fourth larval stage [17].

Two lines of evidence suggest that this programmed cell death is not a normal suicide. First, both B.alapaav and B.arapaav can survive in animals in which the engulfing cell P12.pa has been ablated with a laser microbeam [16]. Second, B.al/rapaav death does not occur in mutants defective in the engulfment genes *ced-1* or *ced-2* [10]. We use “B.al/rapaav” to refer to the homolog that is fated to die (see below), which can be either B.alapaav or B.arapaav. We use “the B.al/rapaav homolog” to refer to the homolog that is fated to survive. These observations suggested that B.al/rapaav death is dependent on P12.pa and that this death might be a murder mediated by the engulfment process. However, it was later observed that B.al/rapaav death also fails in mutants defective in the caspase *ced-3*, leading to the suggestion that this death might be an induced suicide initiated by P12.pa engulfment or by another function of the engulfment genes [12]. We sought to characterize this unusual programmed cell death to further understand the interaction between the cell autonomously-acting cell-death genes and the cell non-autonomous process of engulfment.

## Results

### B.al/rapaav death is caspase- and engulfment gene-dependent

To confirm and extend the observations that B.al/rapaav death is dependent on the engulfment genes *ced-1* and *ced-2* [10] and the caspase *ced-3* [12], we assayed B.al/rapaav death in a variety of cell-death mutant backgrounds. Specifically, we scored the presence of a cell that expressed a reporter specific for the dying B.al/rapaav, *P*_*egl-1*_*::4xNLS::gfp* (see below), in a position consistent with that of an undead B.al/rapaav in late fourth larval stage animals and interpreted such a cell as one that had failed to undergo programmed cell death. In this way, we confirmed that B.al/rapaav cell death is dependent on both the suicide and engulfment pathways. Specifically, strong loss-of-function alleles of the pro-apoptotic genes *egl-1*, *ced-4* or *ced-3* or a gain-of-function allele of the anti-apoptotic gene *ced-9* almost completely blocked B.al/rapaav death (Fig. 1d). Strong loss-of-function alleles of any of the major engulfment genes were also sufficient to prevent B.al/rapaav death (Fig. 1d). In two engulfment-defective animals that we observed into adulthood, the undead B.al/rapaav cell continued to persist for the duration of observation, indicating that engulfment defects prevent B.al/rapaav cell death rather than temporarily delaying it. While loss of engulfment gene function can weakly contribute to the survival of other cells in *C. elegans*, the effect is smaller than that of weak reduction-of-function mutations in suicide genes [12]. By contrast, weak reduction-of-function mutations in the pro-apoptotic genes *ced-3* or *ced-4* had smaller effects on B.al/rapaav survival than did loss of engulfment gene function (Fig. 1d). This finding indicates that B.al/rapaav death is particularly dependent on the function of engulfment genes rather than highly sensitive to any slight perturbation to the cell-death pathway. Mutations in *unc-108* Rab2 cause severe defects in phagosome maturation and cell-corpse degradation after engulfment [18], but a mutation in *unc-108* did not significantly block B.al/rapaav death, suggesting that the engulfment process and not downstream degradation processes are important for B.al/rapaav death.

### The dying B.al/rapaav expresses cell-death genes

**Fig. 2:**
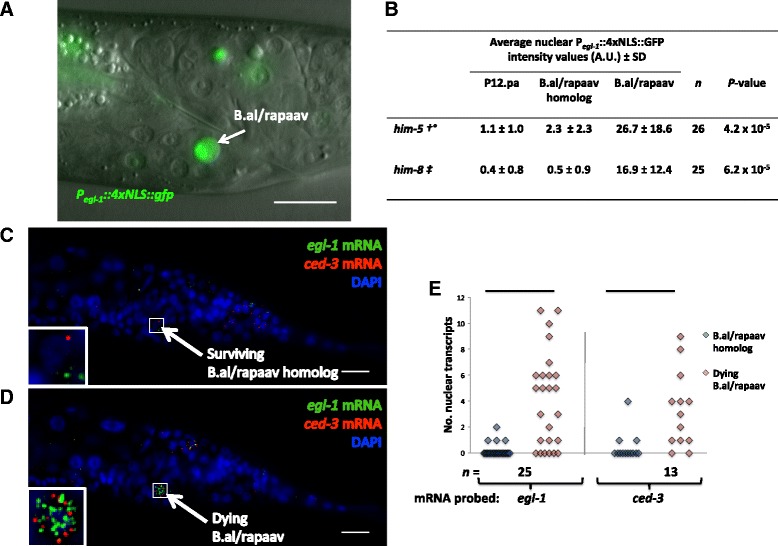
Cell-death genes are expressed in B.al/rapaav. **a** GFP under the control of the *egl-1* promoter is expressed brightly in B.al/rapaav in the tail of an early fourth larval stage “wild-type” male of genotype *nIs343; him-8*. Scale bar: 10 μm. **b** The average intensity of P_*egl-1*_::4xNLS::GFP in the nucleus is higher in the dying B.al/rapaav cell than in the surviving B.al/rapaav homolog in early fourth larval stage animals. The B.al/rapaav fate was assigned based on partial cytoplasmic refractility and/or nuclear position and distance from the midline of the animal. Average fluorescent intensity within the nucleus was measured for confocal images of B.alapaav, B.arapaav and P12.pa in each animal, and the average background intensity was subtracted from each. A.U., arbitrary units. All genotypes include *nIs343*, and some also contain †: *him-5(e1490)*; ‡: *him-8*; °: *nIs349.* **c**–**e** Proapoptotic genes *egl-1* and *ced-3* are not highly expressed in the surviving B.al/rapaav homolog (**c**) but are highly expressed in the dying B.al/rapaav (**d**). These two images were taken from the same animal, approximately 1.6 μm apart. Anterior, left; dorsal, top. Scale bars: 10 μm. Full Z-stack of this animal is available as Additional file 2: Movie 2, and two more examples are available as Additional file 3: Movie 3, Additional file 4: Movie 4. These animals were of the genotype *nIs343; him-5(e1490); nIs349.* **e** Quantification of nuclear mRNA transcripts of *egl-1* and *ced-3* in B.al/rapaav and the B.al/rapaav homolog. Only nuclear transcripts were counted, because we could not unambiguously determine to which cell non-nuclear transcripts belonged. Condensed chromatin (as visualized by DAPI, which stains DNA and shows the chromatin in a dying cell to be slightly brighter and smaller in volume than that in a living cell), a position to the left or right of P12.pa, or expression of *P*_*egl-1*_ *::4xNLS::gfp* was each interpreted as indicating the secondary cell fate. All animals were of the genotype *nIs343*, *him-5(e1490)*, *nIs349.* *: *P* < 0.005, **: *P* < 5 × 10^−4^

The cell-death genes in *C. elegans* act cell-autonomously to specify and cause cell death [3, 9]. To examine if the presumptive cell-death suicide genes indeed act in the dying B.al/rapaav, we used a transcriptional reporter for *egl-1*, the most upstream gene in the core cell-death pathway; *egl-1* is transcriptionally upregulated to drive programmed cell death [4]. The *egl-1* reporter was expressed strongly in the B.al/rapaav cell fated to die and not at all or only weakly in the B.al/rapaav homolog, which is fated to live (Fig. 2a, b). All B.al/rapaav corpses in fourth larval stage wild-type animals were GFP-positive (n = 53), confirming that the GFP expression is associated with the secondary fate of cell death.

We used single-molecule fluorescent in situ hybridization (smFISH) to determine whether, like the *P*_*egl-1*_*::4xNLS::gfp* reporter, endogenous cell-death genes are transcribed in the dying B.al/rapaav. We usually detected *egl-1* mRNA expression in B.al/rapaav and only rarely in the B.al/rapaav homolog, consistent with the expression pattern of *P*_*egl-1*_*::4xNLS::gfp* (Fig. 2c–e and Additional file 2: Movies 2, Additional file 3: Movies 3, Additional file 4: Movies 4). We detected low levels of *ced-9* and *ced-4* transcripts broadly (data not shown). *ced-3* transcripts were visible in only a subset of cells, usually including B.al/rapaav, and only rarely in the B.al/rapaav homolog (Fig. 2c–e and Additional file 2: Movies 2, Additional file 3: Movies 3, Additional file 4: Movies 4). Primary- and secondary-fated cells were identified based on nuclear position and morphology (the secondary-fated B.al/rapaav cell nucleus appears condensed after staining DNA with DAPI to visualize nuclei and is closer to P12.pa). Because B.al/rapaav cell death is dependent on suicide genes that are expressed in the dying B.al/rapaav and not in nearby cells, B.al/rapaav death is likely a form of cell suicide rather than a murder.

### Engulfment genes do not induce the caspase-mediated suicide pathway

**Fig. 3:**
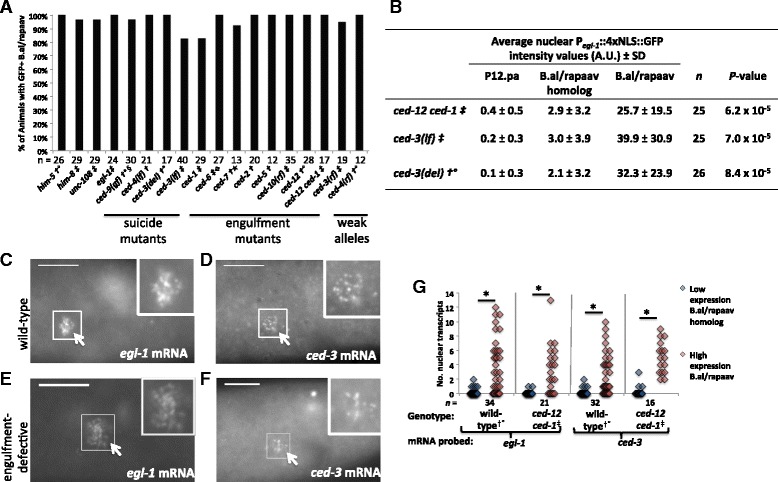
B.al/rapaav and the B.al/rapaav homolog express pro-apoptotic genes differentially in cell-death mutants. All genotypes include *nIs343*, and some also contain †: *him-5(e1490)*, ‡: *him-8*, °: *nIs349*, §: *lon-1(e1820) dpy-17*, : *lon-1(e185)*, ★: *unc-32.* **a** B.al/rapaav expresses *P*_*egl-1*_ *::4xNLS::gfp* in all genetic backgrounds studied. Percentage of animals with *P*_*egl-1*_ *::4xNLS::gfp* expression in death-fated B.al/rapaav during the early fourth larval stage, before B.al/rapaav death occurs. **b** The average intensity of P_*egl-1*_::4xNLS::GFP in the nucleus is higher in B.al/rapaav than in the B.al/rapaav homolog in early fourth larval stage animals defective for cell death genes. A.U., arbitrary units. *egl-1* (**c**, **e**) and *ced-3* (**d**, **f**) transcripts are detectable in the presumptive B.al/rapaav (white arrow) in the tails of early fourth larval stage wild-type (**c**, **d**) and engulfment-defective (**e**, **f**) males. The genotype in **c** was *nIs343; him-8*; **d** *nIs343; him-5(e1490); nIs349*; **e**, **f** *ced-12 ced-1; nIs343; him-8.* Scale bars: 10 μm. **g** *ced-3* and *egl-1* are differentially expressed between B.alapaav and B.arapaav. Only nuclear transcripts were counted. For each animal, the cell with the larger number of transcripts was classified as “high expression B.al/rapaav” and the other cell was classified as “low expression B.al/rapaav homolog.” When both cells had the same number of transcripts, one was arbitrarily labeled high and one low. This panel includes data from Fig. 2e. Animals contained *nIs343* and †: *him-5(e1490)*, ‡: *him-8*, °:*nIs349*. *: *P* < 0.005

As in wild-type animals, *P*_*egl-1*_*::4xNLS::gfp* was expressed in the undead secondary B.al/rapaav in cell-death suicide and engulfment mutants (Fig. 3a, b). Similarly, endogenous transcripts of the cell-death genes *egl-1* and *ced-3* were generally present in one, but not both, of B.alapaav and B.arapaav in the engulfment-defective double mutant, *ced-12 ced-1* (Fig. 3c–g)*.* These data indicate that the induction of suicide gene expression in B.al/rapaav requires neither engulfment nor signals transduced via the engulfment pathway.

**Fig. 4:**
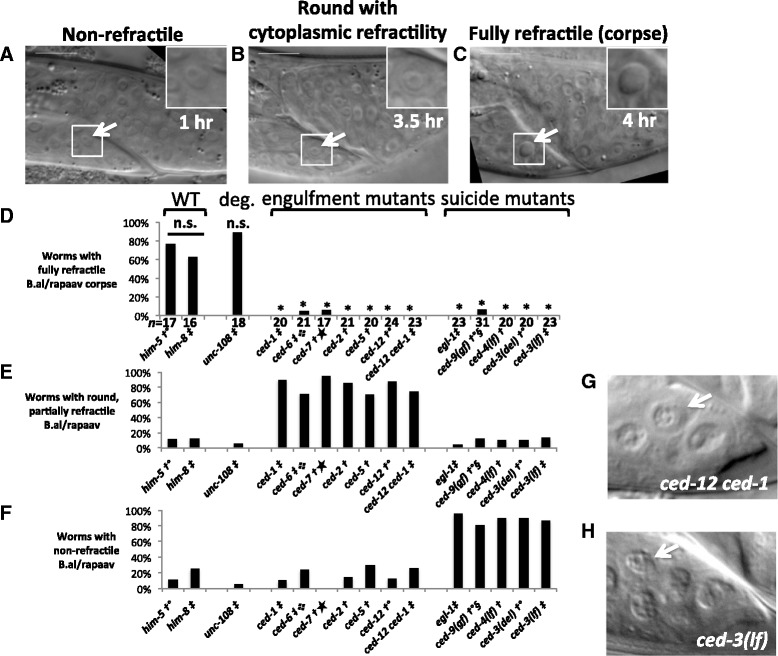
The surviving B.al/rapaav is morphologically different in engulfment-gene and suicide-gene mutants. **a**–**c** The morphology of a dying B.alapaav changes over time; *him-8* fourth larval stage male. Arrows and insets show B.alapaav. Scale bars: 10 μm. **a** 1 hour after its generation, B.alapaav was non-refractile. **b** 3.5 hours after its generation, B.alapaav was rounded and the cytoplasm was refractile, but the nucleus was non-refractile. **c** 4 hours after its generation, B.alapaav was a refractile corpse. **d**–**f** Percentage of animals in which B.al/rapaav displayed the morphology seen in images (**c**), (**b**), and (**a**), respectively, in mid-fourth larval stage animals. Animals with no visible secondary B.al/rapaav, presumably because this cell had already been degraded, were excluded from this analysis. All genotypes include *nIs343* and some also contain †: *him-5(e1490)*, ‡: *him-8*, °: *nIs349*, : *lon-1(e185)*, ★: *unc-32*, §: *lon-1(e1820) dpy-17*. **d** n.s.: *P* > 0.5, *: *P* < 0.005. **d** Wild-type vs. engulfment mutants (*ced-1*, *ced-6*, *ced-7*, *ced-2*, *ced-5*, *ced-12*, and *ced-1 ced-12*): *P* < 10^−17^, wild-type vs. suicide mutants (*egl-1*, *ced-9(gf)*, *ced-4(lf)*, *ced-3(del)*, and *ced-3(lf)*): *P* < 10^−15^, engulfment vs. suicide mutants: *P* > 0.5. **e** Wild-type vs. engulfment mutants: *P* < 10^−11^, wild-type vs. suicide mutants: *P* > 0.5, engulfment vs. suicide mutants: *P* < 10^−31^. **f** Wild-type vs. engulfment mutants: *P* > 0.5, wild-type vs. suicide mutants: *P* < 10^−12^, engulfment vs. suicide mutants: *P* < 10^−30^. *P* values between classes of mutants are by two-tailed Fisher’s exact test using data pooled within genotypic classes (wild-type, engulfment mutants and suicide mutants). **g**–**h** Representative images of the morphology of the undead B.al/rapaav in *ced-12 ced-1* engulfment (**g**) and *ced-3(lf)* suicide (**h**) mutant backgrounds

The dying B.al/rapaav exhibited morphological changes as visualized with Nomarski differential interference microscopy (DIC) similar to those of other dying cells [13, 19]. At the time of their generation, B.alapaav and B.arapaav are indistinguishable using DIC and appear similar to normal healthy cells (Fig. 4a). Later, one (B.al/rapaav) becomes round and condensed with partial cytoplasmic refractility (Fig. 4b) before becoming a highly refractile corpse (Fig. 4c). The other (the B.al/rapaav homolog) remains non-refractile and looks healthy and normal (data not shown).

While mutations in either the suicide pathway or the engulfment pathway were sufficient to block B.al/rapaav from acquiring the highly refractile appearance characteristic of programmed cell death, we discovered that the morphology of B.al/rapaav as viewed with DIC optics was different between these two classes of mutants: in the mid-fourth larval stage, B.al/rapaav was a highly-refractile corpse in most wild-type animals (Fig. 4d); the undead B.al/rapaav in engulfment mutants generally was round with a refractile cytoplasm and non-refractile nucleus (Fig. 4e, g) and the undead B.al/rapaav in *ced-3* and other suicide mutants generally was non-refractile, similar to other living cells, including the B.al/rapaav homolog (Fig. 4f, h). The morphology of the undead B.al/rapaav in engulfment mutants was indistinguishable from that of the dying B.al/rapaav at an earlier stage of the cell-death process. We interpret this morphology to be that of a cell in which caspases have been at least partially activated, as this morphology is dependent on *ced-3* and the rest of the core cell-death pathway. These results further show that the engulfment genes are not required for the initiation of the cell-death process but are rather required in a later process. We examined *unc-108* Rab2 mutants, which are defective in phagosome maturation in the engulfing cell and hence in the degradation of cell corpses [18]. B.al/rapaav died and formed a fully refractile corpse in *unc-108* mutants, suggesting that the failure of B.al/rapaav to form a fully refractile corpse in engulfment mutants is unlikely to be caused by a block in cell-corpse degradation.

**Fig. 5:**
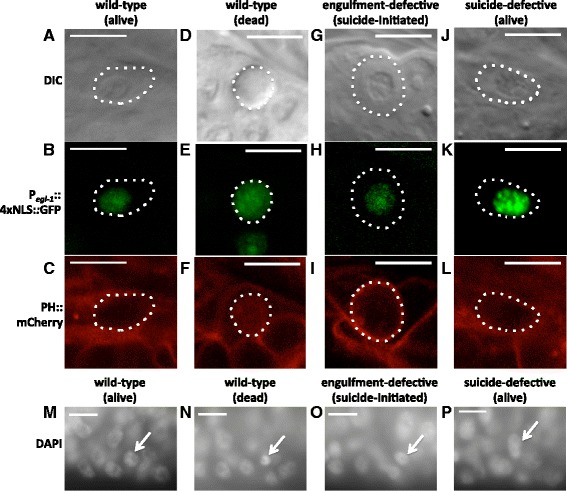
The undead B.al/rapaav cell in engulfment mutants fails to display characteristics of dead cells. **a–c** A living B.al/rapaav cell (cell boundary indicated by dotted line) in the early fourth larval stage before cell death is complete (**a**) lacks refractility in the nucleus, (**b**) has P_*egl-1*_::4xNLS::GFP in the nucleus but not in the rest of the cell and (**c**) has P_*evl-20*_::mCherry::PH localized to the membrane. **d–f** A B.al/rapaav cell corpse has (**d**) refractility throughout the cell, (**e**) P_*egl-1*_::4xNLS::GFP throughout the cell, and (**f**) P_*evl-20*_::mCherry::PH throughout the cell. **g**–**i** An undead B.al/rapaav cell in the late fourth larval stage of an engulfment-defective mutant (**g**) lacks refractility in the nucleus, (**h**) has P_*egl-1*_::4xNLS::GFP in the nucleus but not the rest of the cell, and (**i**) has P_*evl-20*_::mCherry::PH localized to the membrane. **j**–**l** An undead B.al/rapaav cell in the late fourth larval stage of a suicide-defective mutant (**j**) lacks refractility, (**k**) has P_*egl-1*_::4xNLS::GFP in the nucleus but not the rest of the cell, and (**l**) has P_*evl-20*_::mCherry::PH localized to the membrane. Animals were of genotypes *nIs343; him-5(e1467ts) unc-76; nIs349; nEx2344* (**a**–**f**), *nIs343; ced-10 him-8; nIs735* (**g**–**i**), or *nIs343; ced-3 him-8; nIs735* (**j**–**l**). Scale bars: 10 μm. **m** A B.al/rapaav cell (arrow) in the early fourth larval stage has diffuse chromatin similar to neighboring living cells. **n** A dying or dead B.al/rapaav cell in the mid-fourth larval stage has condensed chromatin. **o** An undead B.al/rapaav cell in the late fourth larval stage of an engulfment-defective mutant has diffuse chromatin similar to neighboring living cells. **p** An undead B.al/rapaav cell in a suicide-defective mutant has diffuse chromatin similar to neighboring living cells. Scale bars: 10 μm. Animals were of the genotypes *nIs343; him-5(e1490); nIs349* (**m**, **n**), *ced-12 ced-1; nIs343; him-8* (**o**), or *nIs343; ced-3 him-8* (**p**). **a–l** depict different animals than in (**m**–**p**), since the fixation required for visualizing DAPI staining precludes the use of DIC optics

In engulfment mutants the caspase pathway is activated, but B.al/rapaav death is not completed. The undead B.al/rapaav cell in engulfment mutants is similar to living cells and unlike other unengulfed cells fated to die, which form fully refractile corpses. Like B.al/rapaav cells that have not yet died or undead B.al/rapaav cells in suicide-defective mutants, the undead B.al/rapaav cell in engulfment mutants was not fully refractile by DIC optics, retained nuclear localization of P_*egl-1*_::4xNLS::GFP and membrane localization of the cytoplasmic membrane marker P_*evl-20*_::mCherry::PH (see below), and had diffuse chromatin as revealed by DAPI staining (Fig. 5a–c, g–m, o, p); by contrast, in fully-refractile B.al/rapaav corpses and other cell corpses in the male tail P_*egl-1*_::4xNLS::GFP and P_*evl-20*_*::*mCherry::PH were distributed throughout the cell corpse, and chromatin after DAPI staining appeared condensed (Fig. 5d–f, n). The distribution of P_*egl-1*_::4xNLS::GFP throughout the cell corpse likely reflects nuclear disruption and/or inactivation of nuclear transport; nuclear disruption and chromatin condensation are two hallmarks of apoptotic cell death [20]. Additionally, during time-course observations, we occasionally saw fluctuations in the level of B.al/rapaav cytoplasmic refractility in engulfment mutants, as was previously reported for other cells that had initiated but not completed the cell-death process [12]. These other cells sometimes survived and differentiated, establishing that cells with partial cytoplasmic refractility are not dead. Similarly, B.al/rapaav is not dead, as it can return to a non-refractile morphology and lacks two canonical hallmarks of apoptotic dead cells, nuclear disruption and chromatin condensation. We conclude that *ced-3* activity does not inevitably lead to the death of B.al/rapaav and that the B.al/rapaav cell fated to die in engulfment mutants is abnormal but alive.

### The engulfing cell P12.pa is not required for the B.al/rapaav death

**Fig. 6:**
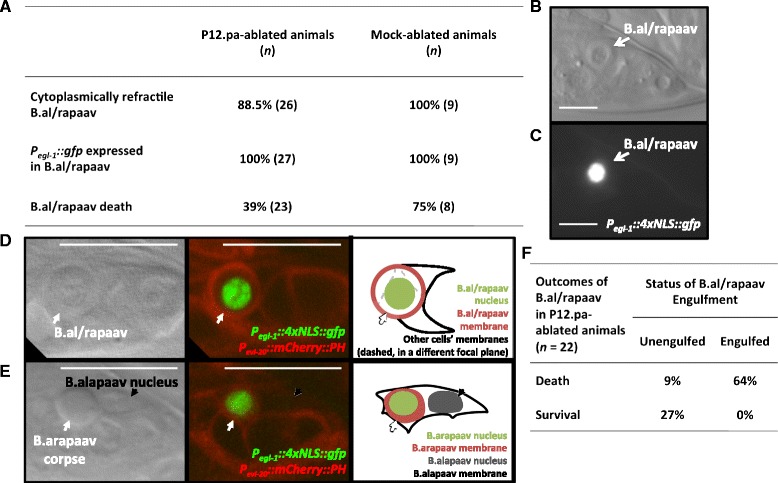
P12.pa ablation does not prevent initiation of B.al/rapaav death. **a** Percentages of P12.pa-ablated and mock-ablated males that have cytoplasmic refractility in B.al/rapaav, *P*_*egl-1*_ *::4xNLS::gfp* expression in B.al/rapaav, or death of B.al/rapaav. Percentage of survival of B.al/rapaav in mock-ablated animals is not significantly different from that of untreated animals in Fig. 1d (*P* = 0.3). **b**, **c** A P12.pa-deficient animal in which the secondary B.al/rapaav (arrow) had cytoplasmic refractility (**b**) and had *P*_*egl-1*_ *::4xNLS::gfp* expression (**c**), indicating that the cell-death process was initiated. Scale bars: 10 μm. All animals in panels (**a**–**c**) were of genotype *nIs343; him-8*. **d** A round, undead B.al/rapaav that was not engulfed in a P12.pa-deficient animal. White arrow points to the undead B.al/rapaav; the only membrane (red) visible around the B.al/rapaav nucleus (green) is that of the undead cell (since only one nucleus is located within that membrane), and no other cell’s membrane appears to enclose it in any plane. Full Z-stack of this animal is available as Additional file 5: Movie 5. **e** A B.arapaav corpse that was engulfed by B.alapaav in a P12.pa-deficient animal. The B.arapaav corpse (white arrow) appears to be completely inside the boundaries of the B.alapaav membrane (black in right panel). This membrane can be assigned to B.alapaav, because it contains the B.alapaav nucleus (black arrow). Full Z-stack of this animal is available as Additional file 6: Movie 6. Scale bars: 10 μm. **f** Outcomes of the secondary B.al/rapaav in P12.pa-ablated animals, showing that all surviving cells were unengulfed while most dying cells were engulfed. All animals in panels (**d**–**f**) were of the genotype *nIs343; him-5(e1467ts) unc-76; nIs349; nEx2344*

It has been reported that the engulfing cell P12.pa is required for B.al/rapaav cell death [16]. To examine the role of P12.pa in B.al/rapaav cell death, we ablated P12.pa or its precursor P12.p early in development, about 15–20 hours prior to the generation of B.alapaav and B.arapaav, and looked for signs that B.al/rapaav had initiated the cell-death program. In the absence of P12.pa, B.alapaav and B.arapaav adopted normal primary and secondary fates and one initiated the cell-death process, as evidenced by *egl-1* reporter gene expression and by the positions and morphologies of the two cells (Fig. 6a–c). Thus, P12.pa is not required for B.alapaav and B.arapaav to differentially adopt the primary and secondary fates or to initiate the death process in the secondary B.al/rapaav.

To our surprise, we found that B.al/rapaav died in many of the animals that lacked P12.pa (Fig. 6a), contrary to the previous report [16]. Thus, although B.al/rapaav death is almost completely dependent on engulfment genes, it is only partially dependent on the presence of the normal engulfing cell. To reconcile this difference, we postulated that other cells might engulf B.al/rapaav in the absence of P12.pa. We designed a reporter protein with mCherry fused to the pleckstrin homology domain, which binds to lipids in the plasma membrane [21], and expressed this construct (*P*_*evl-20*_*::mCherry::PH*) using a promoter from the *evl-20* gene [22], which is widely expressed throughout the animal, including in P12.pa, B.alapaav and B.arapaav (Fig. 6d, e). This construct highlights cell membranes and allowed us to visually determine if a corpse is internalized by another cell. We ablated P12.pa in this strain and found again that B.al/rapaav died in many of the animals. In animals in which B.al/rapaav survived, the undead B.al/rapaav was not engulfed (Fig. 6d, f, and Additional file 5: Movie 5). B.al/rapaav died in 16 P12.pa-ablated animals, and in 14 of these 16 cases the B.al/rapaav corpse was engulfed by a neighboring cell (Fig. 6e, f and Additional file 6: Movie 6). We conclude that the discrepancy between the essentially complete dependence on engulfment genes and the weaker dependence on the engulfing cell P12.pa is a consequence of the compensatory ability of other cells to engulf B.al/rapaav in the absence of P12.pa.

In nine of the 14 animals, the engulfing cell was the surviving primary B.al/rapaav homolog, and in five animals it was a more lateral cell. The identities of the other engulfing cells are uncertain. Based on position, likely candidates are K.a or the left rectal gland cell, B.alapaad and F.lvv. The B.al/rapaav homolog, K.a, the rectal gland cells, B.al/rapaad and F.l/rvv are cells that do not normally engulf corpses, since the only nearby dying cell is B.al/rapaav, which is engulfed by P12.pa. These findings demonstrate that other cells are competent to engulf B.al/rapaav in the absence of P12.pa and that even cells that do not normally engulf cell corpses can have a cryptic ability to recognize and engulf dying cells to promote cell death.

In short, B.al/rapaav likely becomes fated to die and begins to die cell-autonomously but generally requires engulfment by P12.pa or another neighboring cell to fully execute the death process. The death of B.al/rapaav thus does not seem to be an induced suicide, the process proposed by Reddien et al. [12].

### Engulfment by P12.pa precedes the B.al/rapaav death

**Fig. 7:**
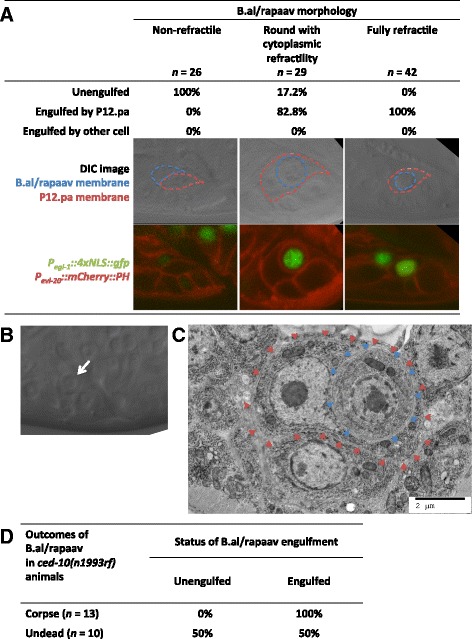
Engulfment precedes the B.al/rapaav death. **a** All non-refractile B.al/rapaav cells are unengulfed, most cells that are round with partial cytoplasmic refractility are engulfed by P12.pa and all fully refractile cells are engulfed by P12.pa. Representative DIC and fluorescent images corresponding to each of the B.al/rapaav morphology classes are shown, with the P12.pa (dashed red line) and B.al/rapaav cell membranes (dashed blue line) outlined (based on P_*evl-20*_::mCherry::PH signal). All animals were in the fourth larval stage, but were at slightly different ages for visualization of B.al/rapaav at different stages of the death process. **b**, **c** A B.arapaav cell with partial cytoplasmic refractility (white arrow) visualized by (**b**) DIC and (**c**) electron microscopy (blue arrowheads). **c** The B.arapaav cell is engulfed by P12.pa (red arrows) but otherwise does not display obvious ultrastructural signs of cell death. The animal was of genotype *him-8.* **d** B.al/rapaav does not die in a weak engulfment mutant without being engulfed. Engulfment and cell-killing are not independent events (*P* = 0.0075, Fisher’s exact test). B.al/rapaav was classified as being dead or undead based on morphology as seen with DIC optics and was then examined to determine whether it was inside of P12.pa based on P_*evl-20*_::mCherry::PH*.* Animals were of the genotype *nIs343; ced-10 him-8; nIs735*

If engulfment is required for the B.al/rapaav death, B.al/rapaav might be engulfed early in the cell-death process, before it becomes a fully refractile corpse. To test this hypothesis, we imaged B.al/rapaav in fourth larval stage animals with cell membranes labeled by P_*evl-20*_::mCherry::PH. We characterized the morphology of each B.al/rapaav as either non-refractile, round with cytoplasmic refractility (suicide-initiated) or fully refractile (corpse). Then, we imaged P_*evl-20*_::mCherry::PH to determine the boundaries of B.al/rapaav and P12.pa. We found that B.al/rapaav corpses were always (42/42) internalized by P12.pa, cell-death initiated cells were usually (24/29) internalized by P12.pa, and cells with no sign of cell death initiation were never (0/26) internalized (Fig. 7a). These data indicate that P12.pa engulfs B.al/rapaav in the early stages of its death, but probably not before the cell death has already been initiated (as evidenced by cytoplasmic refractility). These findings are consistent with our observations that the suicide pathway acts before the engulfment pathway. We also confirmed that the identity of the engulfing cell was always P12.pa in intact animals (n = 66): other cells engulf B.al/rapaav only if P12.pa is absent.

We confirmed that engulfment can precede the completion of cell death using electron microscopy. We observed a dying B.arapaav cell using DIC microscopy and fixed and stained the animal at the stage at which the B.arapaav cell was round with cytoplasmic refractility (Fig. 7b). B.arapaav had already been internalized by P12.pa but, ultrastructurally, it lacked signs of cell death such as nuclear envelope dilation or crenulation, extensive chromatin condensation, and membrane whorling (Fig. 7c) [19].

It is conceivable that engulfment genes act to promote B.al/rapaav cell death by cell-killing activities independent of their roles in engulfment per se. The weak loss-of-function allele *ced-10(n1993rf)* stochastically prevents B.al/rapaav cell death in only some animals (Fig. 1d). We hypothesized that if *ced-10* promoted B.al/rapaav death through a cell-killing activity independent of its role in engulfment, these two functions might stochastically fail in different animals. For example, B.al/rapaav might be killed without being engulfed or engulfed without being killed. However, if the cell-killing activity of *ced-10* were mediated through engulfment, B.al/rapaav would not be killed unless it were engulfed. We found that B.al/rapaav corpses in *ced-10(n1993rf)* were always engulfed by P12.pa (Fig. 7d). Undead B.al/rapaav cells were sometimes unengulfed and sometimes engulfed. These data are inconsistent with the hypothesis that engulfment and cell-killing are independent events (*P* = 0.0075). We conclude that engulfment precedes B.al/rapaav death and that the killing activity of *ced-10* is mediated through its engulfment function.

## Discussion

**Fig. 8:**
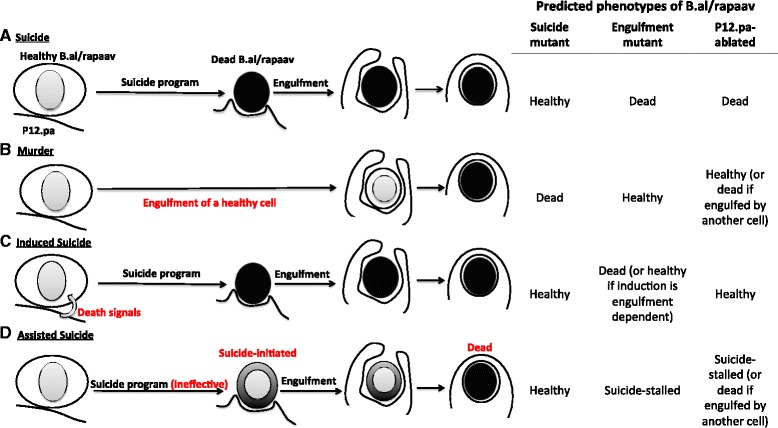
Models of alternative modes of B.al/rapaav cell death. **a** Suicide: B.al/rapaav initiates the suicide program and undergoes programmed cell death, resulting in a fully-refractile corpse, which is engulfed by the neighboring cell P12.pa. A suicide cell death would occur in engulfment mutants and in P12.pa-ablated animals but not in suicide-pathway mutants. Our data are inconsistent with this model, as the death does not occur in engulfment mutants. **b** Murder: B.al/rapaav is healthy until it is engulfed by P12.pa, after which it dies. This form of cell death would occur in suicide-pathway mutants but not in engulfment mutants or P12.pa-ablated animals (unless engulfed and killed by a different neighboring cell). Our data are inconsistent with this model, as the death does not occur in suicide mutants. **c** Induced suicide: B.al/rapaav is signaled by P12.pa to induce the suicide program. This form of cell death would not occur in suicide-pathway mutants or P12.pa-ablated animals but would occur in engulfment mutants (unless the death-inducing signal requires engulfment genes, which is not the case for B.al/rapaav, since the cell-death suicide genes are expressed in engulfment mutants). Our data are inconsistent with this model, as initiation of the B.al/rapaav death process is not dependent on the presence of P12.pa or the activities of the engulfment genes (the cell-death suicide genes are expressed in both cases, and death-related morphological changes can be seen in B.al/rapaav in engulfment mutants and in some animals in which P12.pa was ablated). **d** Assisted suicide: B.al/rapaav initiates the suicide program, which is ineffective at causing programmed cell death and results in a suicide-stalled cell. Once B.al/rapaav is engulfed, the death reaches completion. This form of cell death would not occur in suicide mutants, engulfment mutants, or P12.pa-ablated animals (unless engulfed by another cell). The suicide-stalled cell in an engulfment mutant might show signs of cell-death initiation. Our data are consistent with this model

Like most other cell deaths in *C. elegans*, B.al/rapaav death requires the canonical suicide genes, with these genes being expressed in the cell that dies. Thus, the suicide pathway is activated in B.al/rapaav. However, while such activation is sufficient to kill most cells (Fig. 8a), it is insufficient to kill B.al/rapaav, which appears injured but intact without engulfment. Thus, engulfment is dispensable for other cell deaths but is required for the B.al/rapaav death. This death is unlikely to be a murder (Fig. 8b), as it requires suicide genes, or an induced suicide (Fig. 8c), as neither the engulfing cell P12.pa nor the engulfment genes are required for the initiation of the cell-death process. We conclude that B.al/rapaav death is instead an assisted suicide (Fig. 8d), since engulfment occurs early in the B.al/rapaav death process and is necessary to facilitate the suicide process and cause cell death.

Are there other assisted suicides? Cell deaths in *C. elegans* exist along a spectrum of sensitivities to engulfment. For example, in the anterior pharynx, postdeirid and ventral cord, cells that are fated to die survive only 0–7 % of the time in a *ced-1* engulfment mutant [12], making B.al/rapaav, which survives 96 % of the time (Fig. 1d), a clear outlier. B.al/rapaav death is the only death in *C. elegans* known to be strikingly dependent on engulfment. However, comprehensive studies have not been performed. B.al/rapaav death was identified as possibly engulfment-dependent and further studied in engulfment mutants based on its major dependence on the engulfing cell P12.pa [16]. B.al/rapaav is always engulfed by P12.pa in intact animals. By contrast, Hoeppner et al. [13] found that, for nine of 13 embryonic cell deaths studied, the identity of the engulfing cell varied among animals. Additionally, our studies of B.al/rapaav have shown that cells that do not normally display engulfment activity can have cryptic engulfment ability and engulf the dying cell if the normal engulfing cell is absent. In such cases, ablation of an engulfing cell would not prevent cell death, since a secondary engulfing cell would assume the engulfment function. More comprehensive analysis using engulfment-defective mutants rather than laser-ablation experiments could reveal more instances of assisted suicide in *C. elegans*.

How might engulfment promote the death process? It has recently been reported that engulfment genes promote the death of the sister cell of the *C. elegans* NSM neuron by affecting its precursor cell [23]. Specifically, in the precursor cell that generates the NSM neuron and its sister cell, which dies, there is a higher level of CED-3 activity in the region that will form the dying NSM sister; the engulfment genes *ced-1* and *ced-2* are required for the formation of this gradient, suggesting that engulfment can promote the cell-death process at least in part by causing cells to differentially inherit apoptotic potential. This mechanism is unlikely to be the way in which engulfment causes the B.al/rapaav death. First, the situation is inherently different: B.alapaav and B.arapaav are not sister cells, and at the time of their generation neither is committed to dying [15]. Thus, even if B.alapaav and B.arapaav inherit more pro-apoptotic potential than their sister cells, that potential is not sufficient to cause cell death. Second, engulfment genes are not required for the difference in levels of apoptotic activity in B.al/rapaav and the B.al/rapaav homolog: we have shown that the dying cell B.al/rapaav up-regulates cell-death genes and displays morphological changes characteristic of cell-death initiation while the surviving B.al/rapaav homolog does not do so, and neither of these differences is engulfment-dependent.

Engulfment- or phagocyte-dependent cell deaths have been described in other organisms. For example, phagoptosis of stressed-but-viable neurons in mammals [24] and entosis of weaker-but-viable cells in tumors [25] are engulfment-dependent mechanisms of cell killing. Tumor cells can evade phagocytosis by upregulating the “don’t eat me” signal CD47, and this evasion can be abrogated with anti-CD47 antibodies, which allow tumor cells to be engulfed and killed [26]. However, these deaths appear to be caspase-independent murders rather than caspase-dependent assisted suicides. It was reported that both engulfment and caspase genes are necessary for cell competition in *Drosophila*, a phenomenon in which fitter cells can outcompete and cause the death of neighboring less fit cells [27], but more recent studies suggest that these deaths might not be engulfment-dependent [28]. Purkinje cells in slices of the mouse cerebellar cortex show signs of apoptosis (including caspase-3 activation and TUNEL staining) that is dependent on microglia, which are engulfing cells [29]. However, it is unclear if engulfment is involved or if the microglia act to induce cell suicide by releasing reactive oxygen species. Suicides induced by microglia have been observed in other cases [30, 31].

Do assisted suicides exist in other species? It might be difficult to identify such assisted suicides, since suicide-initiated, stalled cells in *C. elegans* engulfment mutants appear morphologically similar to normal cell suicides in progress. In *C. elegans* it is possible to identify stalled cells as failed deaths because it is known when and where all cell deaths normally occur. It is likely that findings from future studies of assisted suicide in *C. elegans* will provide insights into the cell non-autonomous factors involved in engulfment-dependent deaths of other species. Better understanding of assisted suicides in *C. elegans* could also lead to the identification and study of assisted suicides in other organisms. Given that engulfment-mediated cell deaths likely act in the progression of various human diseases, such as the loss of neurons by phagoptosis in neurodegenerative disorders [24] and the removal of cancer cells through engulfment or entosis [25, 26], further studies of assisted suicides might provide insights of medical importance.

Why does B.al/rapaav require assistance by an engulfing cell to die? B.al/rapaav is able to express *egl-1* and the caspase CED-3 without dying; these processes are necessary but insufficient for the B.al/rapaav death. Weak mutations in *ced-3* or *ced-4* allow other cells to survive only rarely [12], but allow B.al/rapaav to survive most of the time (Fig. 1d). These results suggest that B.al/rapaav is hard to kill and generally resistant to activation of the cell-death suicide pathway, which may be why assistance from an engulfing cell is needed. We believe that CED-3 is activated in the dying B.al/rapaav based on the morphological changes that occur in a caspase-dependent but engulfment-independent manner, but we do not know the level of active CED-3 in B.al/rapaav. Translational and post-translational effectors have been shown to regulate cell death in *C. elegans*, so the level of *ced-3* transcript does not necessarily correspond to the level of activated CED-3 [32, 33]. It is possible that B.al/rapaav has lower levels of activated CED-3 than most dying cells or that B.al/rapaav might require more activated CED-3 to die than most dying cells, and that this difference causes B.al/rapaav death to require assistance. Tumor cells can be more sensitive than non-tumor cells to treatments that induce apoptosis such as radiation and chemotherapy [34]. We suggest that understanding the mechanistic basis of the difference in sensitivity between B.al/rapaav and other cells that undergo apoptosis during *C. elegans* development might reveal aspects of cancer biology.

## Conclusion

We demonstrate that the B.al/rapaav programmed cell death is dependent on both the core apoptotic pathway and the engulfment pathway. Initiation of the cell-death process requires the core apoptotic pathway, but death does not occur unless B.al/rapaav is engulfed. Thus, this death represents a novel form of cell death, which we term “assisted suicide.” This form of cell death might occur in other organisms. Further studies of how engulfment can promote cell death and what makes a cell require non-cell autonomous assistance during the cell-death process will likely reveal findings important for cancer biology and medicine.

## Methods

### Strains and genetics

*C. elegans* was maintained on nematode growth medium (NGM) Petri plates at 20 °C. *him-8(e1489)*, *him-5(e1490)*, and *him-5(e1467ts)* were used as the wild-type backgrounds, because mutations in *him-8* or *him-5* cause hermaphrodites to generate a high incidence of males, which facilitated study of the male-specific cell B.al/rapaav. The following mutations, integrations, and extrachromosomal arrays were used: LGI: *unc-108(nu415)*, *ced-12(n3261)*, *ced-1(e1735).* LGII: *nIs343* [*P*_*egl-1*_*::4xNLS::gfp, lin-15AB(*+*)*]*.* LGIII: *ced-4(n3332lf)*, *ced-4(n3195rf)*, *dpy-17(e164)*, *lon-1(e1820)*, *lon-1(e185)*, *ced-6(n1813)*, *unc-32(e189)*, *ced-7(n1892)*, *ced-9(n1950gf)*. LGIV: *ced-2(e1752)*, *ced-10(n1993rf)*, *ced-5(n1812)*, *him-8(e1489)*, *ced-3(n2427rf)*, *ced-3(n717lf)*, *ced-3(n3692del)*. LGV: *him-5(e1490)*, *him-5(e1467ts)*, *egl-1(n1084 n3082)*, *unc-76(e911)*. LGX: *nIs349* [*P*_*ceh-28*_*::mCherry, lin-15AB(*+*)*]. Extrachromosomal arrays: *nEx2344* [*P*_*evl-20*_*::mCherry::PH, unc-76(*+*)*] (this study). *ts*, temperature-sensitive; *gf*, gain-of-function; *rf*, reduction-of-function; *lf*, total loss-of-function; and *del*, deletion.

### General microscopy

To identify B.alapaav and B.arapaav, we traced their cell lineages in several animals to determine their distinctive positions as previously described [17]. Once it was ascertained that *nIs343* was expressed in the dying B.al/rapaav in all genetic backgrounds studied, we used it as a marker to score the survival of B.al/rapaav in mutant animals. We mounted animals in 20–60 mM sodium azide on 4 % agar pads. We estimated the age of animals based on the following criteria: early fourth larval stage animals had exited the L3/L4 lethargus but had no developed hook structure (approximately 34–37 hours after hatching); mid-fourth larval stage animals had a developed hook structure and the tail was starting to retract (approximately 37–40 hours after hatching); late fourth larval stage animals had visible ray tips or more developed rays but had not entered the period of lethargus between the fourth larval and adult stages (approximately 40–43 hours after hatching). We first used Nomarski differential interference contrast optics to putatively identify B.al/rapaav and categorize its morphology and then confirmed B.al/rapaav identity by checking for *nIs343* expression using a fluorescence-equipped Axioskop II compound microscope (Zeiss, Oberkochen, Germany). Images were acquired with an ORCA-ER CCD camera (Hamamatsu, Hamamatsu City, Japan) using OpenLab software (Agilent, Lexington, MA).

To score engulfment, *P*_*evl-20*_*::mCherry::PH* animals were anesthetized with 60 mM sodium azide and mounted on 4 % agar pads. For data described in Figs. 6 and 7a, animals were imaged at 0.2–0.25 μm intervals in the region containing B.alapaav and B.arapaav using a Zeiss LSM 510 or LSM 800 confocal microscope. For data described in Fig. 7d, P12.pa boundaries were visualized using an Axioskop II compound microscope (Zeiss). A cell was considered to be engulfed if it looked to be within another cell’s boundaries, as visualized by mCherry::PH. Engulfing cell identity was deduced by comparison of nuclear and cell boundary positions to those in the dataset “JSG_male_tail” in the WormImage Database on WormAtlas [35].

Image processing was done using Fiji.

### Single-molecule fluorescent in situ hybridization

Fixation of larval animals, conjugation of fluorescent probes to and purification of oligo probes, hybridization, and imaging were performed as previously described [36]. The *egl-1* set of probes included 21 20-nucleotide probes complementary to regions in the second and third exons and 3’ untranslated region of *egl-1.* The *egl-1* probe set was conjugated to the fluorophore Alexa 594 (Invitrogen, Carlsbad, CA). The *ced-3* set of probes included 48 20-nucleotide probes complementary to regions in all exons of *ced-3*. The *ced-3* probe set was conjugated to Alexa 594 for images and analysis in Figs. 2e, 3d, f, g and to Cy5 (Invitrogen) for Fig. 2c, d. The *ced-4* set of probes included 48 20-nucleotide probes complementary to all exons of the short pro-apoptotic isoform of *ced-4* conjugated to Alexa 594. The *ced-9* set of probes included 48 20-nucleotide probes complementary to all exons and 3’ untranslated region of *ced-9* conjugated to Cy5. Image processing was done using Fiji.

B.alapaav and B.arapaav were identified based on the positions of DAPI-stained nuclei in animals estimated to be between 33 and 37 hours of age based on the progress of the ray lineage cell divisions and deaths. Transcripts within the B.alapaav and B.arapaav nuclei were manually identified and quantified. We analyzed nuclear transcripts because we could not unambiguously determine to which cell cytoplasmic transcripts belonged.

### Laser ablation

First and second larval stage animals were anesthetized with 20 mM sodium azide and mounted on 4 % agar pads. P12.pa was identified by Nomarski differential interference contrast optics and killed using the laser system described by Avery and Horvitz [37]. Mock-ablated animals were mounted along with ablated animals, but the laser was aimed next to the animal. The next day, recovered animals that had reached the fourth larval stage were remounted with food on 4 % agar pads in 10 % polyvinyl pyrrolidone in M9 buffer and checked for normal developmental rate, the absence of P12.pa and the lack of other visible damage before scoring. Slides were sealed with petroleum jelly to prevent drying. Animals were maintained at 20 °C and observed until B.al/rapaav death or until the tips of the rays were visible (late fourth larval stage), at which point they were scored as B.al/rapaav death failing to occur.

### Plasmid construction

To create the *P*_*evl-20*_*::mCherry::PH* transgene, a 3.1 kb fragment 5’ of *evl-20* was PCR amplified using primers with *Pst*I and *Nhe*I restriction sites incorporated on the 5’ ends to facilitate cloning. The purified PCR amplicon was digested with *Nhe*I and *Pst*I, the plasmid pDD111 was digested with *Xba*I and *Pst*I, and the purified digestion products were ligated together (pDD111 is a plasmid containing *P*_*egl-1*_*::mCherry::PH::unc-54 3’ UTR* that was a gift from Dan Denning). An out-of-frame start codon was inadvertently introduced, which was removed by site-directed mutagenesis. The plasmid was injected at 10 ng/μL into *him-5(e1467ts) unc-76(e911)* animals with 50 ng/μL of *unc-76(*+*)* as a co-injection marker.

### Statistical notes

Target sample sizes were selected prior to evaluating significance. Sample sizes varied slightly depending on the number of animals available of the appropriate age for scoring. Data for each experiment were collected over multiple days and pooled. For smFISH experiments, animals were excluded from analysis if the signal was weak in other tissues (e.g. germline for *ced-3* and Rn.aap cells for *egl-1*). To calculate *P* values we used the two-tailed Wilcoxon signed-rank test for quantitative data (i.e. smFISH transcript counts and fluorescence intensity data) and the two-tailed Fisher’s test to compare proportions (i.e. fraction of worms with B.al/rapaav survival, GFP fluorescence or a given morphological appearance). Reported *P* values are corrected for multiple hypothesis testing by the Bonferroni correction. When unspecified, reported *P* values were calculated by comparison with the wild-type strain containing the same *him* mutation.

## Additional files

Additional file 1: Movie 1. B.alapaav and B.arapaav are located close to each other and to the engulfing cell P12.pa in the developing male tail (Z-stack). Figure 1b was traced from the animal shown here. Z-stack scans from right to left; anterior is to the left and dorsal is up. (MP4 9854 kb) Additional file 2: Movie 2. Expression of *egl-1* and *ced-3* in an early fourth larval stage male tail. Figures 2c, d are slices from the animal shown here. Green: *egl-1::*Alexa594. Red: *ced-3::*Cy5. Blue: DAPI. 1 and 2 label the presumptive B.al/rapaav homolog and B.al/rapaav, respectively. Genotype *nIs343; him-5(e1490); nIs349.* Scale bar: 10 μm. Z-stack scans from left to right; anterior is to the left and dorsal is up. (MP4 1575 kb) Additional file 3: Movie 3. Expression of *egl-1* and *ced-3* in an early fourth larval stage male tail. Green: *egl-1::*Alexa594. Red: *ced-3::*Cy5. Blue: DAPI. 1 and 2 label the presumptive B.al/rapaav homolog and B.al/rapaav, respectively. Genotype *nIs343; him-5(e1490); nIs349.* Scale bar: 10 μm. Z-stack scans from left to right; anterior is to the right and dorsal is up. (MP4 2385 kb) Additional file 4: Movie 4. Expression of *egl-1* and *ced-3* in an early fourth larval stage male tail. Green: *egl-1::*Alexa594. Red: *ced-3::*Cy5. Blue: DAPI. 1 and 2 label the presumptive B.al/rapaav homolog and B.al/rapaav, respectively. Genotype *nIs343; him-5(e1490); nIs349.* Scale bar: 10 μm. Z-stack scans from left to right; anterior is to the right and dorsal is up. (MP4 1428 kb) Additional file 5: Movie 5. An undead B.al/rapaav, which was not engulfed, in a P12.pa-deficient animal. Figure 6d is a slice from the animal shown here (note that Fig. 6d was flipped horizontally). Genotype *nIs343; him-5(e1467ts) unc-76; nIs349; nEx2344*. Red: *P*_*evl- 20*_ *::*mCherry::PH. Green: *P*_*egl-1*_ *::*4xNLS::GFP. Scale bar: 10 μm. Z-stack scans from right to left; anterior is to the right and dorsal is up. (MP4 8877 kb) Additional file 6: Movie 6. A dead B.al/rapaav, which was engulfed, in a P12.pa-deficient animal. Figure 6e is a slice from the animal shown here. Genotype *nIs343; him-5(e1467ts) unc-76; nIs349; nEx2344*. Red: *P*_*evl- 20*_ *::*mCherry::PH. Green: *P*_*egl-1*_ *::*4xNLS::GFP. Scale bar: 10 μm. Z-stack scans from left to right; anterior is to the left and dorsal is up. (MP4 3062 kb)
